# Projected future impact of HPV vaccination and primary HPV screening on cervical cancer rates from 2017–2035: Example from Australia

**DOI:** 10.1371/journal.pone.0185332

**Published:** 2018-02-14

**Authors:** Michaela T. Hall, Kate T. Simms, Jie-Bin Lew, Megan A. Smith, Marion Saville, Karen Canfell

**Affiliations:** 1 Cancer Research Division, Cancer Council NSW, Sydney, Australia; 2 School of Public Health, University of Sydney, Sydney, Australia; 3 Victorian Cytology Service Ltd., Melbourne, Australia; 4 Department of Obstetrics and Gynaecology, University of Melbourne, Melbourne, Australia; Universidade Estadual de Maringa, BRAZIL

## Abstract

**Background:**

Many countries are transitioning from cytology-based to longer-interval HPV screening. Trials comparing HPV-based screening to cytology report an increase in CIN2/3 detection at the first screen, and longer-term reductions in CIN3+; however, population level year-to-year transitional impacts are poorly understood. We undertook a comprehensive evaluation of switching to longer-interval primary HPV screening in the context of HPV vaccination. We used Australia as an example setting, since Australia will make this transition in December 2017.

**Methods:**

Using a model of HPV vaccination, transmission, natural history and cervical screening, *Policy1-Cervix*, we simulated the planned transition from recommending cytology every two years for sexually-active women aged 18–20 to 69, to recommending HPV screening every five years for women aged 25–74 years. We estimated rates of CIN2/3, cervical cancer incidence, and mortality for each year from 2005 to 2035, considering ranges for HPV test accuracy and screening compliance in the context of HPV vaccination (current coverage ~82% in females; ~76% in males).

**Findings:**

Transient increases are predicted to occur in rates of CIN2/3 detection and invasive cervical cancer in the first two to three years following the screening transition (of 16–24% and 11–14% in respectively, compared to 2017 rates). However, by 2035, CIN2/3 and invasive cervical cancer rates are predicted to fall by 40–44% and 42–51%, respectively, compared to 2017 rates. Cervical cancer mortality rates are predicted to remain unchanged until ~2020, then decline by 34–45% by 2035. Over the period 2018–2035, switching to primary HPV screening in Australia is expected to avert 2,006 cases of invasive cervical cancer and save 587 lives.

**Conclusions:**

Transient increases in detected CIN2/3 and invasive cancer, which may be detectable at the population level, are predicted following a change to primary HPV screening. This is due to improved test sensitivity bringing forward diagnoses, resulting in longer term reductions in both cervical cancer incidence and mortality. Fluctuations in health outcomes due to the transition to a longer screening interval are predicted to occur for 10–15 years, but cervical cancer rates will be significantly reduced thereafter due to the impact of HPV vaccination and HPV screening. In order to maintain confidence in primary HPV screening through the transitional phase, it is important to widely communicate that an initial increase in CIN2/3 and perhaps even invasive cervical cancer is expected after a national transition to primary HPV screening, that this phenomenon is due to increased prevalent disease detection, and that this effect represents a marker of screening success.

## Introduction

With the introduction of HPV vaccination, and improvement of screening technologies for the secondary prevention of cervical cancer, several countries are planning a programmatic transition from cytology-based cervical screening to primary HPV screening.[1–4] Reductions in HPV associated disease due to the types protected against by vaccination have been extensively documented, following the widespread implementation of HPV vaccination in many countries.[5–7] A quadrivalent HPV vaccine is currently used in the Australian National HPV Vaccination program.[8] This protects against four HPV types: 6 and 11 (implicated in >90% of anogenital warts [9,10]) and types 16 and 18 (implicated in ~70–80% of all cervical cancers[11,12]). In 2007, the National HPV Vaccination Program was implemented in Australia targeting females aged 12–13, with an additional catch-up program for ages 13–26 in 2007, which was completed at the end of 2009, implemented via schools and primary care.[13] Males aged 12–13 years have been included in the program since 2013, and catch-up vaccination for males aged 14–15 years was offered until the end of 2014.[14,15] Data released by the National HPV Vaccination Program Register in Australia indicates high three-dose coverage rates in 2014 (78% in females and 72% in males).[15] Reductions in infections associated with HPV 16/18, anogenital warts and high-grade cervical precancer are well documented, including in unvaccinated individuals due to herd protection effects.[5–7,16–20]

Australia is imminently transitioning, on December 1^st^ 2017, from the current National Cervical Screening Program (‘pre-renewed NCSP’) involving two-yearly (once every two years) cytology to five-yearly (once every five years) primary HPV screening in late 2017 (‘renewed NCSP’).[21] Australia’s transition to primary HPV screening is underpinned by a detailed review of the evidence and a modelled analysis of a range of screening test technologies conducted by the government’s Medical Services Advisory Committee in 2013.[22,23] The Cancer Council Australia Cervical Cancer Prevention Guidelines Working Party was formed in 2015 to develop detailed guidelines for the management of HPV-positive women in the new program, and these were finalised in 2016.[21] Our previous modelled analyses for Australia have shown that five-yearly primary HPV screening for women aged 20–74 years is more effective than cytology at shorter intervals (i.e. intervals of two-three years).[23,24]

The pre-renewed NCSP in Australia, introduced in 1991, recommends that asymptomatic sexually-active women aged 18–20 to 69 years attend for routine conventional cytology testing every two years.[25] From December 2017, in the renewed NCSP, it will be recommended that women aged 25–74 years attend for primary HPV testing every five years, with partial genotyping for HPV 16 and 18, and liquid based cytology (LBC) triage for women in whom oncogenic HPV (not 16/18) is detected. Women in whom HPV 16/18 is detected, or women whose triage LBC is reported as ASC-H (atypical squamous cells, possible high grade lesion) or worse after testing positive for oncogenic HPV (not 16/18) will be referred for colposcopy, while other women with a positive HPV test will be referred for follow-up with an HPV test in 12 months. This is based on (i) evidence suggesting that women who are positive for HPV types 16 and/or 18 are at a higher risk of developing CIN2+ than those with oncogenic HPV types not 16/18 (when other oncogenic types are taken as a group)[22], and (ii) the inclusion of HPV 16/18 in vaccines leading to lower prevalence of these types in young women in the post-vaccine era, thus opening up a practical strategy for HPV screening starting at age 25 years which includes partial genotyping with immediate referral to colposcopy for these types.[26,27]

Randomised controlled trials indicate that switching from cytology to primary HPV screening will result in increased CIN2/3 detection in early screening rounds (particularly the first round); because detected CIN2/3 is treated, this reduces CIN2/3 and invasive cervical cancer in subsequent screening rounds.[28–30] Hence, transitional effects are likely to occur in screening programs after the implementation of a more sensitive test, and also because of the change in screening interval.[28] Our recent study indicates that transitional outcomes in screening and diagnostic test volumes will fluctuate significantly in the few years following the transition from cytology to primary HPV screening in Australia, due to the longer interval.[31] However, the transitional impacts of a changing screening program on health outcomes, such as high-grade cervical disease rates, cervical cancer incidence and mortality rates has not been previously estimated. In light of this, here we aimed to predict the short and long-term impact of such a transition on health outcomes at the population level, accounting for screening history and vaccine impact. Using Australia as an example, we aimed to estimate year-by-year rates of CIN2/3, and incidence and mortality rates for invasive cervical cancer, over the period from 2005 to 2035, taking into account the impact of the National HPV Vaccination Program and the transition to primary HPV screening in late 2017.  

## Materials and methods

### *Policy1-Cervix* model platform

We utilised a comprehensive model platform of cervical cancer natural history, cervical screening, HPV transmission, and vaccination (‘*Policy1-Cervix’*), which has been extensively calibrated and validated in a range of settings.[24,31–40] The platform consists of a dynamic model of HPV transmission and vaccination (implemented in Microsoft Visual Studio C++), coupled with a deterministic Markov model of the natural history of HPV and cervical screening and invasive cervical cancer survival (implemented using TreeAge Pro 2014, TreeAge Software, Inc., MA, USA). This model platform has been used to evaluate the effectiveness and cost-effectiveness of the renewed NCSP for both unvaccinated and vaccinated cohorts in Australia, and informed the development of the guidelines for the Renewed NCSP.[21,23] In the current analysis, the model simulated multiple birth cohorts, from ages 0 to 84 years, accounting for age-specific rates of benign hysterectomy and other-cause mortality. Outcomes in these cohorts were used to produce cross-sectional outcomes in women for the period 2005–2035. A complete description of calibration targets has been reported elsewhere.[23] Further information about the model platform can be found in S1 Appendix.

### Modelling of cervical screening

**Fig 1:**
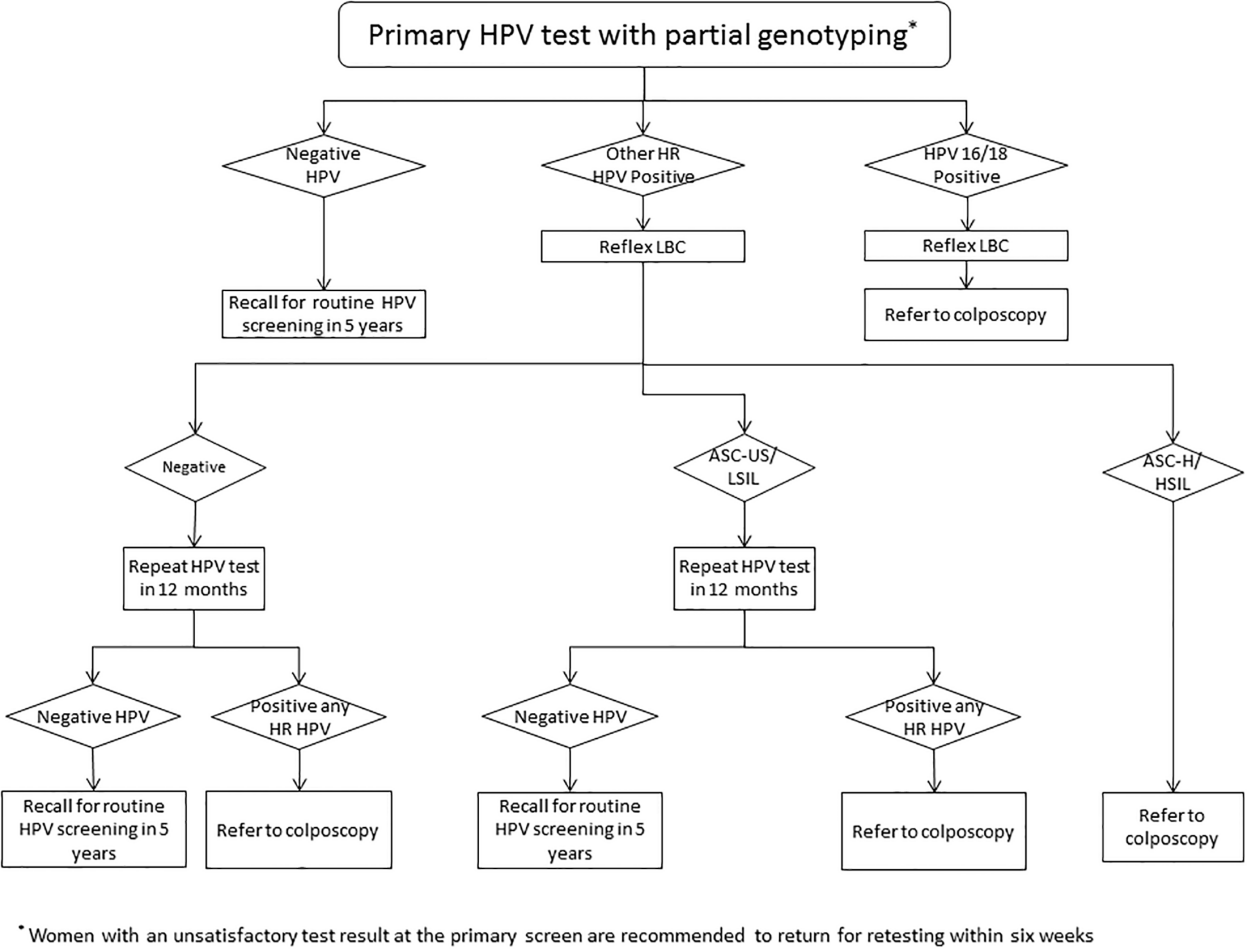
Primary HPV testing with partial genotyping and cytology triage management flow chart.

The pre-renewed NCSP was modelled based on 2005 National Health and Medical Research Council (NHMRC) Guidelines.[41] Detailed structure, assumptions and data sources used have been previously described.[23] Briefly, the pre-renewed NCSP recommends two-yearly screening with conventional cytology for asymptomatic sexually-active women aged 18–20 to 69 years. Compliance rates for screening and follow-up in the pre-renewed NCSP were based on data from the Victorian Cervical Cytology Registry (VCCR), which includes all cervical cytology tests undertaken in the state of Victoria (~25% of the Australian population) who have not opted off the registry.[42,43] The renewed NCSP was modelled accounting for the latest national cervical screening program guidelines (see Fig 1).[21] In the model, five-yearly primary HPV screening is simulated with HPV 16/18 genotyping and LBC triage (manually-read) for women aged 25–74 years in whom oncogenic HPV types (not 16/18) are detected. Women in whom triage LBC is reported as ASC-H or worse are referred for colposcopy, as are women in whom HPV 16/18 is detected. Women in whom oncogenic HPV (not 16/18) is detected and whose triage LBC is reported as negative, ASC-US (atypical squamous cells, undetermined significance) or LSIL (low-grade squamous intraepithelial lesion) are referred for 12 month follow-up with HPV testing. Additionally, women who test HPV negative at 12 months receive an invitation to rescreen in five years, while those in whom oncogenic HPV (any type) is detected at 12 months are referred to colposcopy.

When modelling the transition from pre-renewed NCSP to renewed NCSP, due to model simulating annual time-steps, women received a primary HPV test from 2018. The model assumes that women under 25, even if they had already initiated screening prior to 2018, would not return for cervical screening until their 25^th^ birthday. Women who were under surveillance for a previous abnormality, regardless of their age, were assumed to switch-over to HPV testing management, as per the new management guidelines.[21]

### Model parameters, assumptions and data sources

The key model parameter assumptions are summarised in Table 1. Model assumptions have been previously described in detail elsewhere and in S1 Appendix.[23]

**Table 1:** Baseline assumptions for key model parameters, and data sources.

Model parameters	Baseline assumption	Data source
Unsatisfactory rate of conventional cytology	2.10%	Australian Institute of Health and Welfare (AIHW) 2013 [44]
Test accuracy of conventional cytology for CIN2+ (ASC-US threshold)	Sensitivity: 74.1%; Specificity: 95.7%	Fitted to AIHW 2013 [45]
Test accuracy of HPV testing for primary screening ^a^	Sensitivity: 96.4%; Specificity: 90.1%	Derived from pooled sensitivity and specificity reported in Arbyn et al. 2012 [46]
Test accuracy of HPV testing for follow-up women treated for high-grade CIN ^a^	Sensitivity: 93.2%; Specificity: 80.8%	Arbyn et al. 2012 [46]
Unsatisfactory rate of manually-read LBC	1.80%	Based on estimates of unsatisfactory rates of image-read LBC in an Australian setting *Davey et al*. *2007* [47] and other studies showing that image-read LBC and manually-read LBC are similar *Bolger et al*. *2006*.[48]
Test accuracy of manually-read LBC for CIN2+ (ASC-US threshold)	Sensitivity: 77.0%; Specificity: 94.7%	Arbyn et al. 2008 [49], S1 Appendix
Test accuracy of HPV partial genotyping for CIN2+ (ASC-US threshold)	100% accuracy in differentiating between HPV 16/18 and non-16/18 infections among women who test positive for any oncogenic HPV types	Assumption.
Colposcopy positive rate	No CIN: 50.2%; CIN1: 76.5%; CIN2+: 88.4%	Data from Royal Women’s Hospital in Victoria.
Screening initiation rate ^b^	*Pre-renewed NCSP*: Proportion of women who have participated in screening at least once by age of 20, 25, 30, 40 and 69 was 32%, 74%, 85%,95% and 98%, respectively. *Renewed NCSP*: assumed a screening initiation invitation is sent on a woman’s 25th birthday and is associated with a rapid screening uptake rate (74%) at 25 years; the proportion of women who have participated in screening at least once by the age of 30 or older remain the same as per *Pre-renewed NCSP*	Data from VCCR in Victoria [23]
Routine screening compliance (pre-renewed NCSP) ^c^	Two-yearly reminder system (calibrated to age-specific participation rates over two, three and five years). Re-attendance rate by two, five and seven years since last screening event was 53%, 94% and 96%, respectively.	Data from VCCR in Victoria [23,50]
Routine screening (transitional period)^d^	No active recall—current system of reminders only (at 27 months) continues. Re-attendance therefore follows same pattern as for pre-renewed NCSP: cumulative re-attendance by 2, 5 and 7 years since last screening event was 53%, 94% and 96%, respectively.	Data from VCCR in Victoria [23]
Routine screening compliance (renewed NCSP) ^c^	Five-yearly call-and-recall system assumed a very low number of early re-screeners and high number of on-time screeners; re-attendance rate by three, five and seven years since last screening was <1%, 86% and 93% respectively.	Creighton et al 2010 [23,50]
Compliance to a recommendation to return in 12 months (follow-up management)	The compliance rate was 65–82% by 12 months and 72–94% by 24 months if previous screening outcome was high-grade abnormal; 62–83% and 80–94%, respectively, if previous screening outcome was low-grade abnormal; 42–53% and 57–86%, respectively, if previous screening outcome was normal. The modelled compliance rates vary by age group and previous screening outcome.	Data from VCCR in Victoria [23]
Age-specific compliance to colposcopy referral	82–96% (variation dependent on age and the reason for referral i.e. low/high-grade cytology etc.)	Data from VCCR and Royal Women’s Hospital in Victoria.
Stage-specific survival assumptions for symptomatically detected cervical cancer	The modelled five-year survival was 80.9% for localised cancer, 61.7% for regional cancer, and 27.9% for distant cancer.	Kang 2012 [51]
Relative survival for screen-detected cervical cancer vs. symptomatically-detected cancer	Localised cervical cancer: 1.15; regional/distant cervical cancer: 1.17	van der Aa et al. 2008 [52], Zucchetto et al. 2013 [53], Andrae et al. 2012 [54]
Vaccination coverage rate	Uptake rates were obtained for each year from 2007–2011 from the NHVPR. We assumed effective coverage occurred at the midpoint of age-specific two- and three-dose coverage data from the NHVPR, and additionally are adjusted for known under-reporting of doses to the NHVPR in catch-up cohorts of females.[14] Effective vaccination coverage for those aged 12 or younger from 2012 onwards is 82.3% in females and 75.5% in males.	NHVPR 2014 [55], NHVPR 2017 [15], Brotherton et al 2008 [56], Brotherton et al 2011 [57], Brotherton et al 2014 [14]

AIHW-Australian Institute of Health and Welfare; CIN- cervical intraepithelial neoplasia; NHVPR—National HPV Vaccination Program Register; VCCR—Victorian Cervical Cytology Registry

^a^ For CIN2+ detection

^b^ Screening participation rate among women who have never participate in the screening program before.

^c^ Screening re-attendance rate among women who have participated in the screening program at least once before.

^d^ Re-attendance for first routine screening test (HPV test) after NCSP transitions

### Compliance with screening and follow-up tests

The modelled age distribution of screening initiation and behaviour under the pre-renewed NCSP were informed by an analysis of data on screening behaviour and follow-up and management compliance over a 10-year period from the VCCR. Modelled five-yearly routine screening compliance under a call-and-recall system, as per the renewed program, was informed by rates of on-time and early attendance as observed in England (another setting which utilises a call-and-recall system), scaled so the proportion of under-screened (still had not re-attended by seven years) matched rates observed in the Australian state of Victoria.[50] Re-attendance following recommendation to return for follow-up in 12 months were assumed to remain the same as in the pre-renewed NCSP. Compliance assumptions, including those for five-yearly routine screening, follow-up and colposcopy attendance under the renewed-NCSP have been detailed previously and can be found in Table 1. [23]

### Vaccination assumptions

The dynamic HPV transmission model accounts for the effect of both direct and indirect protection from HPV vaccination in different birth cohorts over time. Vaccine uptake rates were modelled based on the midpoint of observed two- and three-dose coverage rates in females since the vaccination program was implemented from the National HPV Vaccination Register, as well as coverage rates in the female catch-up program and male vaccination (Table 1, S1 Appendix).[55–57] Detailed vaccine coverage assumptions can be found in S1 Appendix. We assumed that the HPV vaccine is only effective in individuals not currently infected with HPV, and that it provides lifelong protection against HPV16/18 but no cross-protection against other oncogenic HPV types; these are simplifying assumptions. We assumed ongoing use of quadrivalent vaccine in the National HPV Vaccination Program.

### Scenarios

To identify the impacts of HPV vaccination and the changes to the screening program, we estimated rates and case numbers for histologically-confirmed CIN2/3, cervical cancer incidence and mortality under the following four scenarios: the base case (realistic) scenario assumed a transition from the pre-renewed NCSP to the renewed NCSP in 2018, taking into account the impact of the HPV vaccination program; Counterfactual scenario 1 assumed no change to the current NCSP and no vaccination; Counterfactual scenario 2 took into account the impact of the HPV vaccination program but assumed no change to the current NCSP; and Counterfactual scenario 3 assumed a transition from the pre-renewed NCSP to the renewed NCSP in 2018 but no vaccination. The base case scenario therefore reflects the best predictions of future outcomes, but other scenarios were modelled in order to unpack the various influences on these outcomes. Further information about specific management assumptions relating to the pre-renewed NCSP and the renewed NCSP can be found in S1 Appendix. Note that our predictions were based on the timing of the program transition being December 2017.

### Main outcomes

Age-standardised rates and case numbers were estimated for each year from 2005–2035, for CIN2/3, invasive cervical cancer diagnosis, and cervical cancer mortality. Histologically-detected CIN2/3 rates were estimated per 1,000 women screened and per 1,000 women in the population. While it is common practice to present these rates per 1,000 women screened, in this analysis we focused on CIN2/3 detection rates per 1,000 women so we may directly compare between scenarios and years, which we have previously estimated will have vastly different numbers of women screened.[31] The period of 2005–2035 was selected in order to visualise cervical disease trends prior, to as well as after, the switchover. Age-standardisation used the 2001 Australian Standard Population released by the Australian Bureau of Statistics (ABS).[58] Case numbers were based on ABS estimates of the resident population from 2005–2012 (2018 projected female population size 12,588,425)[58] and ABS ‘Series B’ population projections from 2013 to 2035.[43]

For the base case scenario (incorporating the effect of both HPV vaccination and the renewed NCSP) we produced detailed results for CIN2/3 histology, invasive cervical cancer incidence and cervical cancer mortality stratified by age, attributable HPV type i.e. HPV 16/18 versus oncogenic HPV (not 16/18), and stage at diagnosis (for invasive cancer).

We estimated the cumulative lifetime risk (CLR) of cervical cancer mortality for cohorts born from 1971–2025 for the base case scenario. We considered women born in 1971 because they were the first cohort eligible to receive the benefit of organised screening from age 20 in Australia, and provided predictions for women born as late as 2025 to incorporate the impact on cumulative lifetime risks across all birth cohorts. All health outcomes were estimated for women aged 0–84 years.

### Sensitivity analysis

Sensitivity analysis on the multiple-cohort outcomes for the base case scenario was conducted (Table 2). Previous modelled analyses have indicated that HPV test sensitivity and screening compliance assumptions are influential on the overall outcomes.[59] Therefore these parameters were considered in a (univariate) sensitivity analysis. In particular, we considered a scenario where the cross-sectional sensitivity of HPV screening for invasive cancer present at the time of screening, was reduced to 81% (extreme assumption). The results of these sensitivity analyses were then presented alongside baseline results to indicate the range of expected uncertainty.

**Table 2:** Sensitivity analysis ranges for key model parameters.

Model parameters	Baseline assumptions	Parameters considered for sensitivity analysis
HPV test positive rate	CIN2 detection: 93% CIN3+ detection: 98.4%	Lower range: CIN2 detection: 89%; CIN3 detection: 95.9%; Invasive cervical cancer detection: 81% (extreme assumption)
Screening coverage assumption (five-yearly screening strategy only)	Baseline (See Table 1)	Lower range: Higher early re-screening: five-yearly call-and-recall system (presentation of 30–33% early rescreening; 62–66% cumulative on time screening, rescreening by 7+ years same as biennial scenario)

## Results

**Fig 2:**
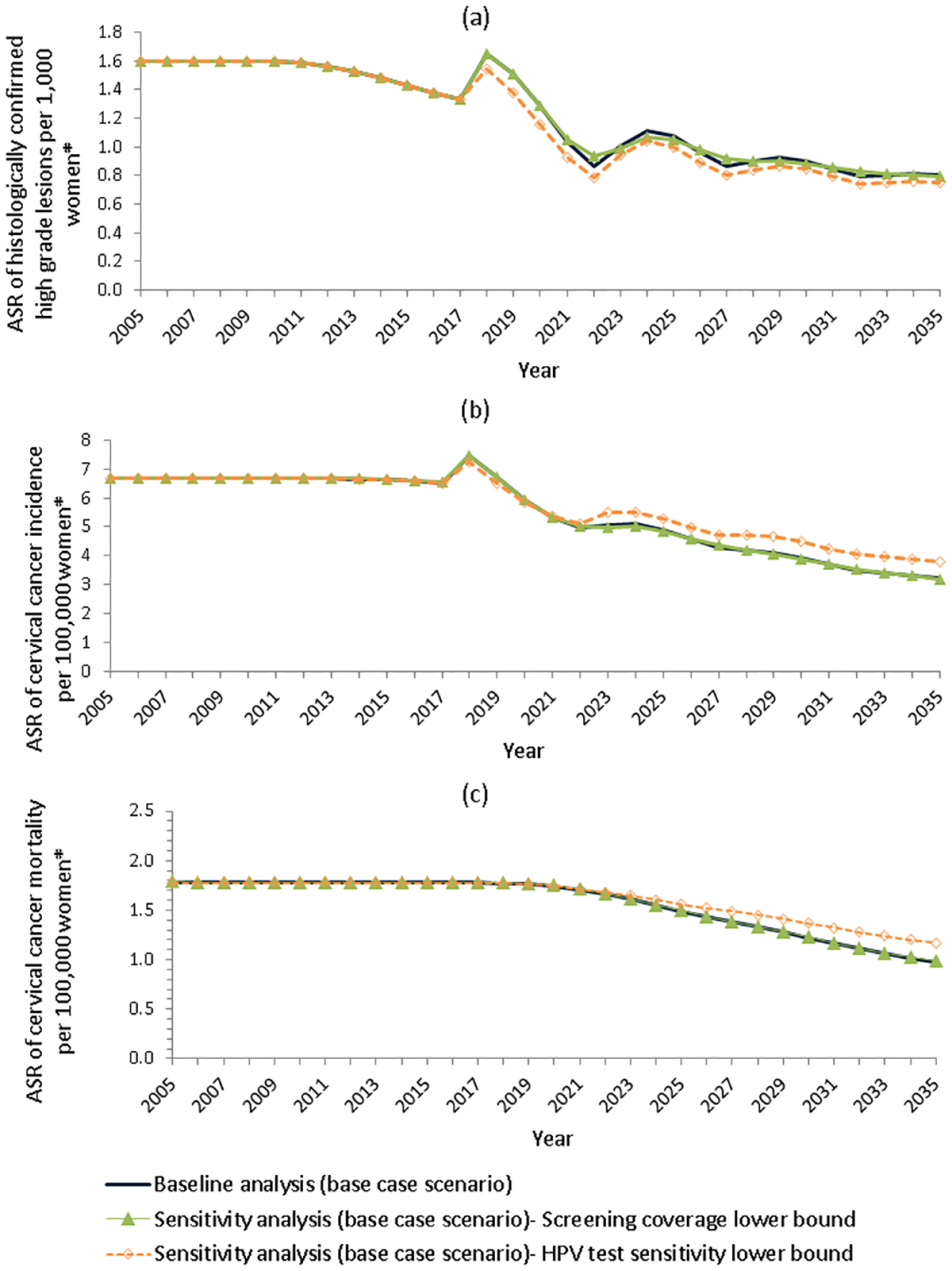
Predicted age standardised rates (ASR) for: (a) CIN2/3 per 1,000 women, (b) cervical cancer diagnosis per 100,000 women and (c) cervical cancer mortality per 100,000 women; base case scenario shown. *Ages considered are 0–84 years; age standardised rates are standardised to the Australian Bureau of Statistics 2001 ‘Series B’ population estimates.

For the scenario incorporating HPV vaccination and the transition to the renewed-NCSP (base case scenario), we predicted that in the first screening round (i.e. in 2018), age-standardised rates of CIN2/3 detection per 1,000 women will increase from 1.33 in 2017 to 1.65 (range: 1.55–1.65), a relative increase of 24% (range: 16–24%). Similarly, in the first round, cervical cancer incidence (per 100,000 women) will increase from 6.52 in 2017 to 7.46 (range: 7.25–7.61), a relative increase of 14% (range: 11–14%). That is, an additional ~4,084 histologically-detected CIN2/3 cases and ~129 invasive cervical cancer cases are expected in 2018 compared to 2017. However, by 2035, decreases are predicted in age-standardised rates of CIN2/3 detection (40% decrease; range: 40–44%)), invasive cervical cancer incidence (51%; 42–51%) and cervical cancer mortality (45%; 34–45%), compared to 2017 rates. Similarly, in 2035 ~3,958 fewer histologically-detected CIN2/3, ~239 fewer invasive cervical cancer diagnoses and ~40 fewer deaths caused by cervical cancer are predicted, compared to 2017. Ranges are obtained using results from the sensitivity analyses, and presented with the result from baseline analysis in Fig 2.

**Fig 3:**
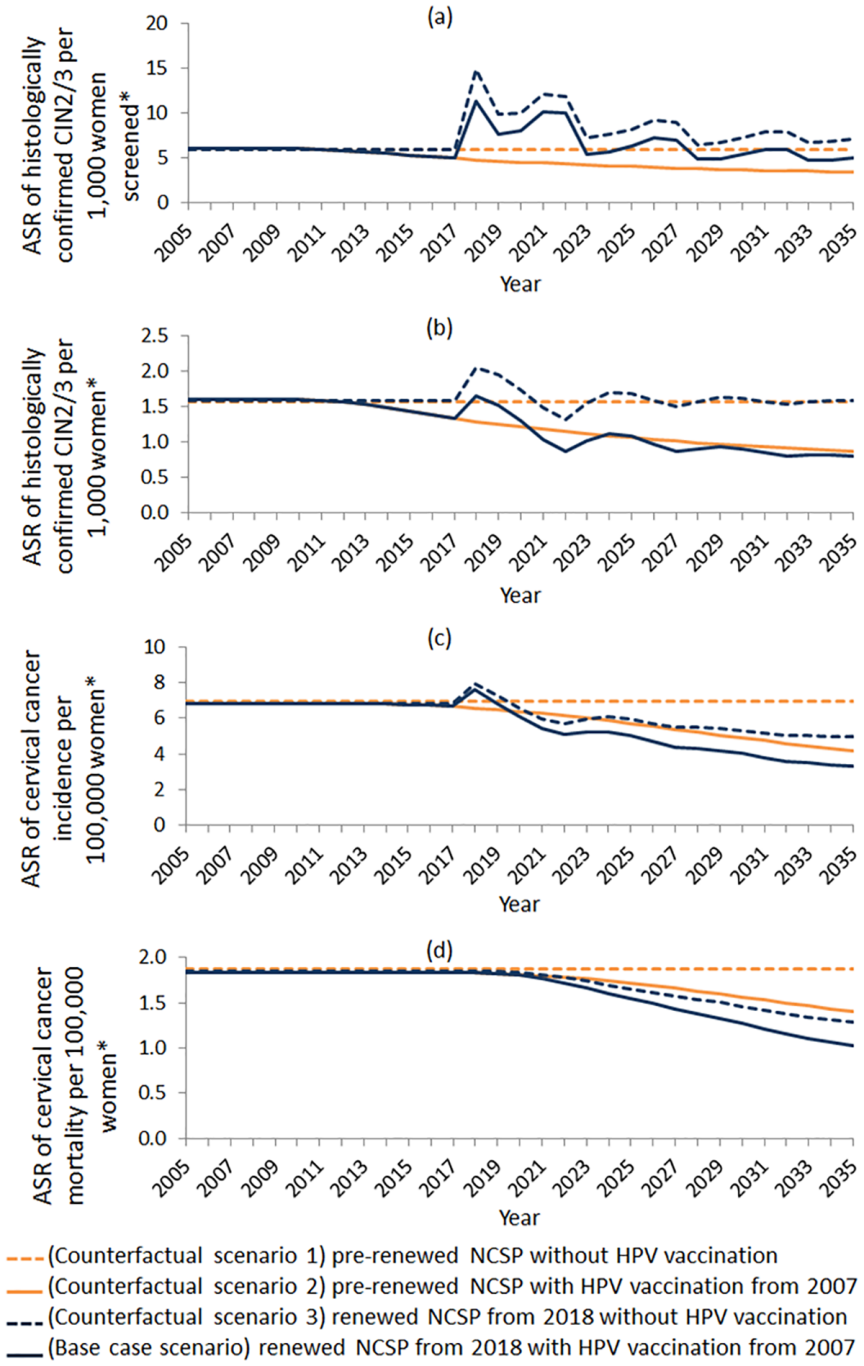
Predicted age standardised rates (ASR) for: (a) CIN2/3 per 1,000 women and (b) 1,000 women screened, (c) cervical cancer diagnosis per 100,000 women and (d) cervical cancer mortality per 100,000 women; base-case and counterfactual scenarios 1–3 are shown. *Ages considered are 0–84 years; age standardised rates are standardised to the Australian Bureau of Statistics 2001 ‘Series B’ population estimates.

The independent impact of HPV vaccination and screening on disease outcomes, age standardised rates of histologically-confirmed CIN2/3, invasive cervical cancer incidence and cervical cancer mortality over a 20-year time interval, from the screening transition, are shown in Fig 3. Fluctuations in disease detection rates following implementation of the renewed NCSP are attributable to a change in screening interval, and the long term decreases due to HPV vaccination are also evident.

Rates of CIN2/3 detection per 1,000 women screened are predicted to be higher from 2018 to 2035 under the renewed NCSP than they would be if cytology-based screening were to continue (Fig 3a), due both to the longer screening interval and thus fewer screening events overall as well as the increased detection rate with primary HPV screening. Fig 3b implies that the initial fluctuations in CIN2/3 detection per 1,000 women following the transition to the renewed NCSP in the base case scenario will dampen and eventually drop below rates predicted for the scenario assuming continuation of the pre-renewed NCSP (Counterfactual Scenario 2) from 2025. This is somewhat earlier than would have been expected in the absence of HPV vaccination (see Fig 3b Counterfactual Scenario 1 vs Counterfactual Scenario 3). Notably, over the period 2018–2035, switching to the renewed NCSP is expected to avert 2,006 cases of invasive cervical cancer and save 587 lives.

**Fig 4:**
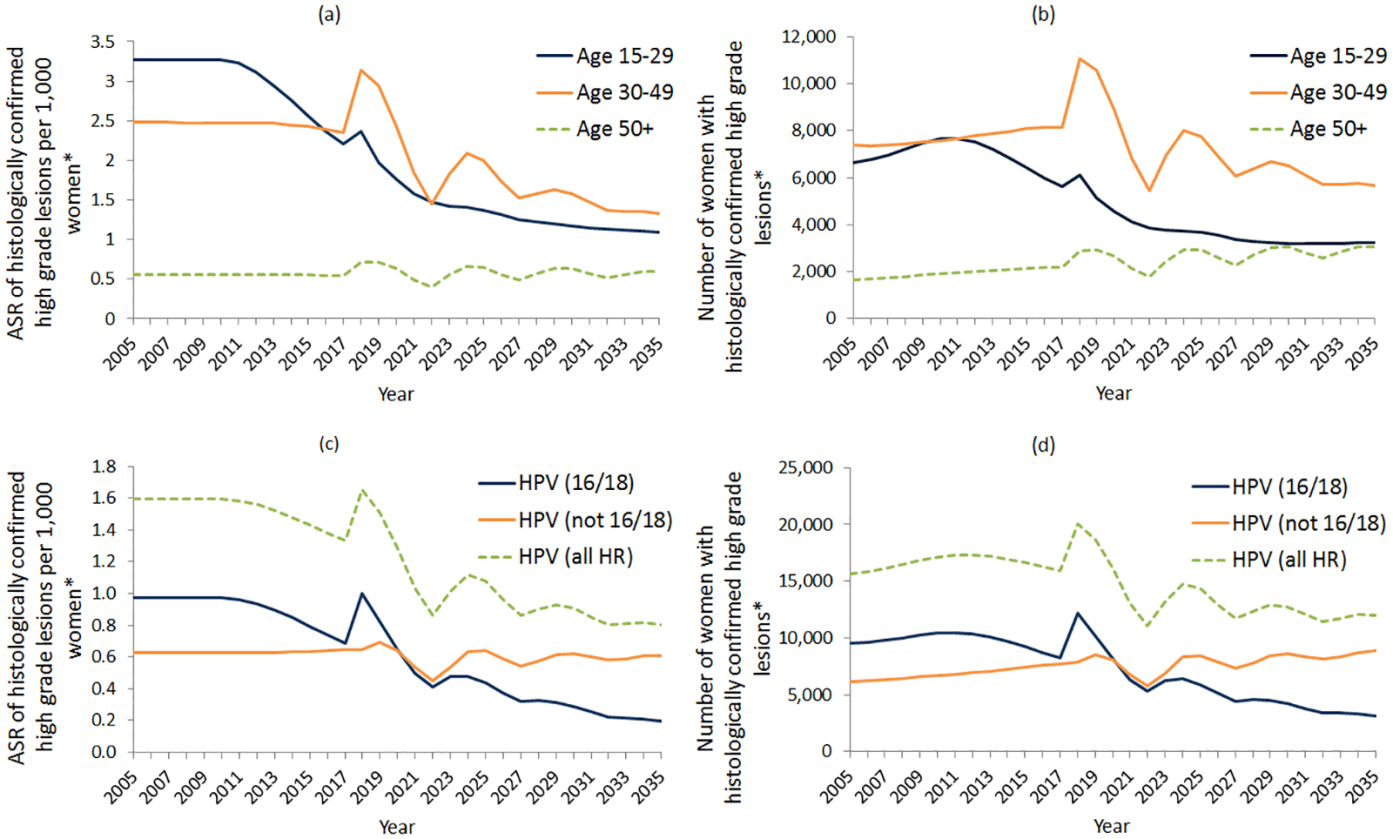
(a) ASR per 1,000 women and (b) case numbers of histologically detected high grade cervical abnormalities by age group, presented with (c) ASR and (d) case numbers of histologically detected high grade cervical abnormalities by HPV type; base case scenario shown. *Ages considered are 0–84 years; age standardised rates are standardised using the Australian 2001 Standard Population; case numbers are calculated using the Australian Bureau of Statistics ‘Series B’ population estimates.

As illustrated in Fig 4, fluctuations in CIN2/3 detection in the base case scenario are present in all age groups 30–49 years and 50+, and, for all attributable HPV types. In women aged under 30 years, fluctuations in histologically-detected CIN2/3 rates (per 1,000 women) and cases reach equilibrium sooner than any other age group, and this age group experiences the greatest decrease in detected abnormalities. We observed a similar phenomenon in women aged 30–49 years, except fluctuations have greater magnitude and the decreasing trend will be less pronounced. Notably, in 2035 in women aged 50 years and older, an overall increase of ~53% in rates of histologically-detected CIN2/3 cases is predicted (compared to 2017 rates) where transitional fluctuations are less severe (Fig 4a and 4b).

During the first round of primary HPV screening starting in 2017, a transient spike is predicted in CIN2/3 attributable to HPV 16/18 and (to a lesser extent) oncogenic HPV (not 16/18) (Fig 4c). Rates per 1,000 women are predicted to increase by 46% and 0.3%, respectively, in 2018, compared to 2017, and subsequently fluctuate with the screening interval. The amplitude of fluctuation will gradually decline over time for HPV (16/18) and HPV (not 16/18) attributable disease. Both the number of cases and the rates of HPV (16/18) attributable CIN2/3 are predicted to decrease over time, but this is not predicted to occur for CIN2/3 attributable to HPV (not 16/18), which will become proportionally more important in the post-vaccination era.

**Fig 5:**
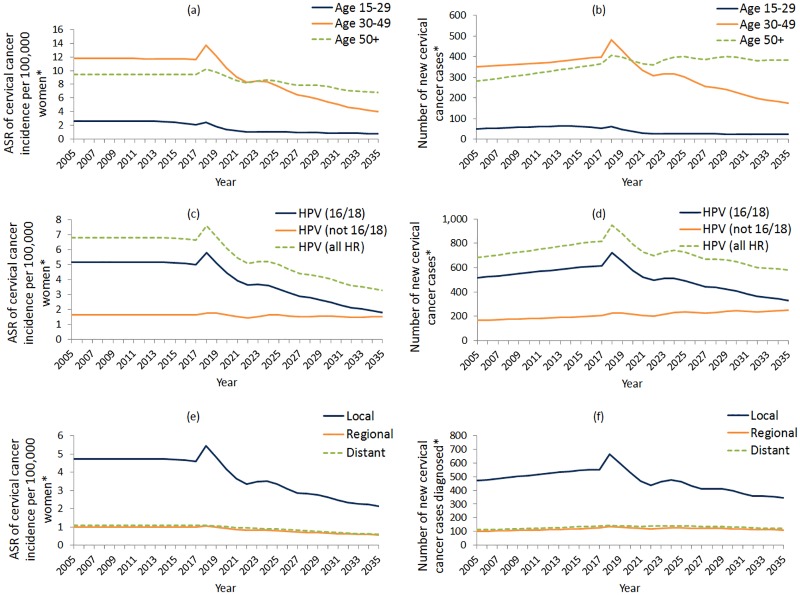
(a) (c) (e) Age standardised rates and (b) (d) (f) case numbers of cervical cancer incidence by age group, HPV type and stage at diagnosis; base case scenario shown. *Ages considered are 0–84 years; age standardised rates are standardised using the Australian 2001 Standard Population; case numbers are calculated using the Australian Bureau of Statistics ‘Series B’ population estimates.

Stratifying cervical cancer incidence rates and case numbers by age, a transient increase, followed by a decrease, is predicted for both age groups 15–29 years and 30–49 years, with the largest decrease in the 30–49 age group (Fig 5a and 5b). For women aged 50 and over, while rates of invasive cervical cancer diagnosis are predicted to decrease over time, case numbers are predicted to continue to increase in this timeframe, in line with population growth. Using 2017 as a benchmark, the age standardised rates of invasive cervical cancer incidence in women aged <30 years, 30–49 years, and 50+ years are predicted to decrease by 62%, 66% and 29% respectively by 2035.

Fig 5c and 5d show predicted trends in invasive cervical cancer incidence by attributable HPV-type. A transient increase in HPV (16/18) attributable invasive cervical cancer is predicted following the first round of 5-yearly primary HPV screening. However, we predict a 65% reduction in HPV (16/18) attributable invasive cervical cancer by 2035, as compared to 2017 rates. In contrast, rates of invasive cervical cancer attributable to HPV (not 16/18) appear to stabilise quickly with an 8% reduction compared to 2017 rates.

As depicted in Fig 5e and 5f, the transient increase in invasive cervical cancer detection following the switchover in 2018 appears to be attributable to an increase in localised disease. Thereafter, gradual decrease is predicted in localised cervical cancer incidence over time, with a 54% reduction in rates of localised invasive cervical cancer by 2035 as compared to 2017 rates. Incidence rates and cases of invasive cervical cancers detected at regional or distant stage are expected to be stable throughout the switchover, and then decrease over time by 43% and 46%, respectively, compared to 2017 rates.

Cervical cancer mortality rates are predicted to steadily decline, from about 2020, following the transition to the renewed NCSP, driven by the continued effects of vaccination and screening (Fig 3d). The percentage reduction in cervical cancer mortality rates in 2035, as compared to 2017, is predicted to be 65% for women aged 15–29 years, 65% for women aged 30–49 years and 29% for women aged 50+.

**Fig 6:**
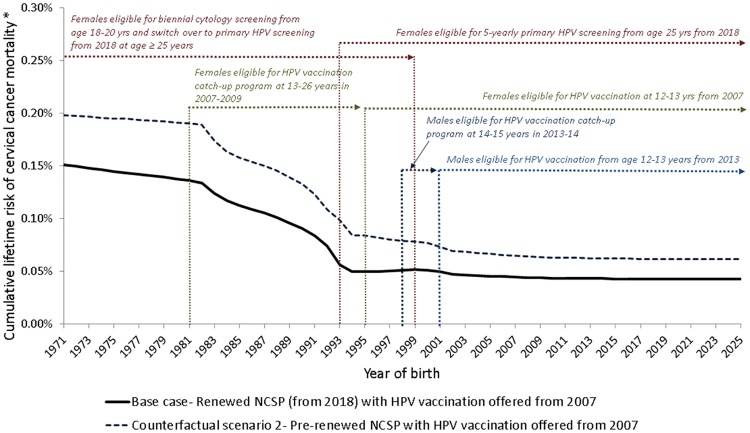
Modelled cumulative lifetime risk of cervical cancer mortality by birth year of base case scenario presented with timing of milestones in cervical cancer prevention initiatives. * Cumulative lifetime risk is calculated to age 84 years.

Fig 6 presents the CLR of cervical cancer mortality for each birth cohort of women from 1971 to 2025, under the renewed NCSP (base case scenario) versus pre-renewed NCSP (counterfactual scenario 2), taking into account HPV vaccination. Under the renewed NCSP, all birth cohorts represented in Fig 6 are exposed to the renewed NCSP at some point in their lives, with the oldest group (born in 1971) modelled as transitioning at age 47 in 2018. By switching over to the renewed NCSP at 47 years instead of continuing in the pre-renewed NCSP, this age group will reduce their cumulative lifetime mortality risk by 24%. Cohorts born in 2025 are predicted to have a 31% lower CLR of cervical cancer mortality under the renewed NCSP compared to the pre-renewed NCSP. Cohorts born later than 1981 are also predicted to have a substantially lower risk as a result of HPV vaccination.

## Discussion

To our knowledge, this is the first study to evaluate the implications on health outcomes of a transition to longer interval HPV testing on a programmatic scale, and to provide year-by-year estimates of health outcomes for more than three screening rounds after the transition from cytology to longer-interval HPV based screening. We found that in the year following the transition to the new primary HPV screening program, rates of histologically-detected CIN 2/3 and cervical cancer will increase by 24% (range: 16–24%;~4,083 additional cases) and 14% (range:11–14%;~129 additional cases) respectively. Rates of high-grade histology and cervical cancer detection will continue to fluctuate, with CIN2/3 eventually falling by 40% (range: 40–44%) and cervical cancer incidence rates falling by 50% (range: 42–51%) by 2035 as compared to pre-renewed NCSP rates. The predicted decrease of HPV (not 16/18) attributable cancer in the simulated timeframe is lower than that predicted for HPV types 16/18. This is to vaccine impact on HPV 16/18; the overall patterns over time are also linked to the differences in management for women positive for HPV 16/18 vs non-16/18 oncogenic types. The transient increase in cervical cancer incidence and CIN2/3 after the first round of screening is in line with clinical trial observations, in which an increase in CIN2/3 detection is observed after the first round of screening due to improved test sensitivity.[29,30] The predicted transient increase in cervical cancer detection was driven by a predicted increase in detecting localised cancers. Due to the continued long-term favourable effect of HPV vaccination and the renewed NCSP, cervical cancer mortality rates were predicted to decline over time, with a reduction in cervical cancer mortality rates of 44% by 2035.

There are many strengths to this study. We separately reported rates of cervical cancer incidence and high-grade histology detection assuming no impact of vaccination, no change in cervical screening, and for a scenario in which no changes are predicted to occur, which meant we could separate out the impacts of HPV vaccination and the change in cervical screening program. We used an extensively calibrated and validated multiple-cohort model of dynamic HPV transmission, vaccination, HPV natural history and cervical screening, which took into account detailed vaccination coverage rates as observed in Australia, the impact of herd effects and screening compliance rates as reported in registry data. Modelling of the new screening program was based on expert advice and the detailed management guidelines in the 2016 recommendations for the renewed cervical screening program.[21]

As for all modelled analyses, the predictions here are contingent on our assumptions and should thus be interpreted with some caution. The current analysis is based on currently available evidence, and explorations into the effect of future changes to screening participation, the National HPV Vaccination Program regime, vaccine uptake or cervical cancer treatment are outside of the scope of this analysis (these are the subject of ongoing work). Screening behaviour is assumed to remain constant over time apart from adapting to the renewed NCSP recommendations. This is a significant limitation because 1) women may change their screening behaviour leading up the switchover, 2) participation among currently under-screened women may improve as a consequence of them being able to access screening via self-collection [5], and 3) following the implementation of HPV vaccination, screening compliance rates in young vaccinated women began to fall.[60] However, this may be mitigated by the increase in the recommended starting age for screening, the use of active invitations, and the implementation of a renewed program which explicitly considered optimal screening in the context of HPV vaccination.

Another limitation is that the modelled analysis may underestimate the impact of HPV vaccination, for three distinct reasons. Firstly, we assumed no cross-protection against non-vaccine-included types; although some evidence shows that HPV vaccines provide a degree of cross-protection against HPV types 31, 33, 45, and 58, the long-term duration of cross-protection at a population level is more uncertain.[16,61] Secondly, we assumed no vaccine efficacy in currently infected women- i.e. that women infected with an HPV type at the time of vaccination, receive no additional protection from vaccination. Although this may further increase actual vaccine impact in catch-up cohorts it should be borne in mind that for ongoing vaccination of 12–13 years old’s only a very small proportion are expected to have been exposed to HPV at the time of vaccination. Thirdly, type replacement could potentially (theoretically) result in an apparent increase in the rate of high-grade lesions and cancer cases attributed to non-vaccine included types, although there is no evidence of this occurring at the population level in countries with high vaccination coverage.[5,62] Overall, our assumptions are conservative with respect to vaccine impact, and thus our estimates of the degree of the fluctuations caused by the screening program transition (which are dampened by vaccination impact) may be considered to be ‘worst case’.

As a fourth limitation, we assumed use of the quadrivalent HPV vaccine for the cohorts modelled. At the time of this analysis, the quadrivalent vaccine was used in the National HPV Vaccination Program (NVHP), and this has been used for 11 years (since the implementation of HPV vaccination in 2017). However, a switch to HPV9, using a two-dose schedule, was recommended by Australia’s Pharmaceutical Benefits Advisory Committee in August 2017.[63] Vaccinating future cohorts with HPV9 is expected to further reduce disease rates to lower levels by additionally providing protection against HPV types 31/33/45/52/58. Although HPV9 introduction in Australia has not been announced, and the timing is still uncertain, it will only impact cohorts turning 12 years of age in 2018 at the earliest. This cohort will not enter the screening program until 2031, and thus the introduction of HPV9 will have minimal impact on our results overall—it will only act to slightly increase vaccine impact in the last 4 years of our analysis period. Introducing a two-dose regime may also affect vaccine coverage in these younger cohorts, because it is expected to facilitate higher coverage delivery in the population overall. However, the extent of this effect is unknown, as the spacing between the two doses must be at least five months, and early data suggest that most females who received fewer than three doses in the current program have a spacing of less than five months between doses.[64] Nevertheless, the current analysis already incorporates an assumption of uptake which is higher than observed three-dose uptake, in order to account for some degree of protection from fewer than three doses (consistent with data from a number of studies).[64–67]. Additionally, the vaccine uptake assumed in the current analysis is already relatively high (~82% in females and ~76% in males), and potentially approaching the point where additional increases may have limited incremental effect, based on a meta-analysis of findings from 16 transmission models.[68] Screening recommendations may also differ for these younger cohorts offered HPV9: for example, our recent analysis suggests that only twice-lifetime screening (at ages 30 and 40) would remain cost-effective in Australian cohorts offered the vaccine.[69]

Another limitation of this analysis is that it does not account for possible losses in vaccine confidence where vaccine coverage rates decrease significantly. For example, this type of event has been observed in Denmark, where HPV vaccine coverage rates have fallen.[70] While such an event in Australia would be expected to increase disease rates above predicted levels, once again it unlikely that this would substantially affect our findings, since any cohorts where this could occur are not highly represented in this analysis. Our modelled analysis does not account for additional measures that may be implemented in the renewed-NCSP, such as targeting under-screened populations by implementing a self-sampling program.[33] Finally, we assume that there are no changes to cervical cancer survival over the next 20 years, as cancer survival in Australia has been relatively unchanged since around 1991.[71] If cervical cancer treatment were to improve, associated mortality rates would be further decreased in Australia below the rates predicted in this analysis. These areas, and related issues, will be further informed by emergent data over time. For example, emerging data from the Compass trial, which is a large scale clinical trial comparing 2.5 yearly cytology screening with 5-yearly HPV screening is expected to play an important role in confirming and refining our findings.[72]

Our study has important implications both for health services planning, and for public communications about the transitional impact of the screening change. Most notably, it is important that screening policy-makers, providers and women are prepared for a transient increase in detection of high grade cervical abnormalities and cervical cancer; and that it is communicated that this represents a success of screening (increased detection with a more sensitive test) and not a failure. It is important to communicate that outcomes for women will continue to improve because of the innovations represented by HPV vaccination and HPV screening.

## Supporting information

S1 Appendix Additional methodological details and steady-state analysis. (DOCX)
