# Supplementary material for: Projected future impact of HPV vaccination and primary HPV screening on cervical cancer rates from 2017–2035: Example from Australia
Source: PLoS One. 2018 Feb 14;13(2):e0185332. doi: 10.1371/journal.pone.0185332 (PMC5812553; doi:10.1371/journal.pone.0185332)
Supplement: S1 Appendix — (DOCX) [file pone.0185332.s001.docx]

Projected future impact of HPV vaccination and primary HPV screening on cervical cancer rates from 2017-2035: Example from Australia

S1 Appendix: Additional methodological details and steady-state analysis

Michaela T Hall^1^, Kate T Simms^1¶,^ Jie-Bin Lew^1¶^, Megan A Smith^1, 2,^ Marion Saville^3, 4,^ Karen Canfell^1, 2*^

1. Cancer Research Division, Cancer Council NSW, Sydney Australia

2. School of Public Health, University of Sydney, Sydney Australia

3. Victorian Cytology Service Ltd., Melbourne Australia

4. Department of Obstetrics and Gynaecology, University of Melbourne, Melbourne Australia

* Corresponding author

E-mail: karen.canfell@nswcc.org.au

¶These authors contributed equally to this work.

# Model platform description

The model platform used in our evaluation, ‘*Policy1-Cervix’*, is a dynamic model of sexual behaviour, HPV transmission and HPV vaccination which has been implemented in Microsoft Visual Studio C++. This is overlayed with a deterministic semi-Markov model of the natural history of CIN development, progression to invasive cervical cancer and cervical screening and follow-up management; which was developed using TreeAge Pro 2014 (TreeAge Software, Inc., MA, USA).

‘*Policy1-Cervix’* has, under a different name, been extensively validated in a range of settings, and utilised to estimate outcomes of cervical screening in a range of countries. The reader is encouraged to find a detailed description of model calibration targets [[1](#_ENREF_1)].

# Screening management and scenarios description

## Pre-renewed NCSP

Screening scenarios which are described as ‘pre-renewed NCSP’ refer to a program offering 2-yearly cytology (Pap) testing for asymptomatic women aged 18-20 to 69 years. In these scenarios, we assume that women with symptoms or screen-detected cervical abnormalities receive follow-up care with repeat cytology, or, colposcopy referral with biopsy (if indicated) depending on her age and screening history, as informed by the 2005 NHMRC Guidelines. [[2](#_ENREF_2)]

Women with negative cytology screening result are continue with 2-yearly routine screening; women with low-grade cytology result are referred to a 12 months repeat cytology testing unless they have a low-grade or worse cytology result in the past 24 months, older than 30 years or have no history of negative cytology in the past 24 months, in which case, they are referred for colposcopy assessment; all women with high-grade cytology result are referred to colposcopy assessment.

## Renewed NCSP

The ‘renewed NCSP’ refers to a program which offers five-yearly primary HPV screening with partial genotyping for asymptomatic women aged 25-74 years. Under this scheme women who are positive for high risk HPV types other than 16 or 18 receive LBC triage; whereas women with low grade or negative cytology are referred for a repeat HPV test in 12 months, and, women with high grade cytology are referred directly for colposcopy. Women positive for HPV types 16 or 18 are also referred directly for colposcopic evaluation. Further downstream considerations are consistent with the draft clinical guidelines for the renewed NCSP. [[3](#_ENREF_3)]

In scenarios which consider a transition from the pre-renewed NCSP to the renewed NCSP in 2017, we assume that all women 25 years and over will receive an invitation to attend for routine screening under the new program, provided they had not been screened in the previous year. At this routine visit, they receive primary HPV testing with partial genotyping in place of cytology. Following a women’s first primary HPV test, we assume that she is managed under the active call-and-recall system whereby an invitation to screen will be sent before she is due for her next screening test.

# Detailed vaccination coverage assumptions

In the baseline analysis we assume vaccine coverage rates in 12-13 year old females since the program was implemented in 2007 including is in the vicinity of 72%; we also consider women up to age 26 who were offered HPV vaccination as part of a catch-up program, as informed by the National HPV Vaccination Register (2014). [[4](#_ENREF_4),[5](#_ENREF_5)] Male vaccination was assumed to start in 2013 for males ages 12-13 (catch-up for 14-15 year old males from 2013 to 2014), and the baseline analysis assumed coverage rates in males is equivalent to female rates. [[6](#_ENREF_6)] Table S1 and Table S2 display detailed cumulative vaccination rate assumptions for females and males, respectively, by age and calendar year; both the baseline scenario, and upper-bound vaccination scenario (as considered in sensitivity analysis) are presented.

Table S1 Detailed vaccination coverage assumptions in females by age and calendar years considered in baseline analysis

|  | ***Calendar year*** | | | | | | |
| --- | --- | --- | --- | --- | --- | --- | --- |
| ***Age (years)*** | ***2007*** | ***2008*** | ***2009*** | ***2010*** | ***2011*** | ***2012*** | ***2013 onwards*** |
| ***12*** | 0% | 77% | 76% | 76% | 78% | 82% | 82% |
| ***13*** | 0% | 77% | 0% | 0% | 0% | 0% | 0% |
| ***14*** | 0% | 77% | 0% | 0% | 0% | 0% | 0% |
| ***15*** | 77% | 76% | 0% | 0% | 0% | 0% | 0% |
| ***16*** | 74% | 0% | 0% | 0% | 0% | 0% | 0% |
| ***17*** | 68% | 0% | 0% | 0% | 0% | 0% | 0% |
| ***18*** | 21% | 0% | 0% | 0% | 0% | 0% | 0% |
| ***19*** | 17% | 23% | 0% | 0% | 0% | 0% | 0% |
| ***20*** | 17% | 21% | 28% | 0% | 0% | 0% | 0% |
| ***21*** | 16% | 20% | 24% | 0% | 0% | 0% | 0% |
| ***22*** | 16% | 19% | 22% | 0% | 0% | 0% | 0% |
| ***23*** | 16% | 19% | 22% | 0% | 0% | 0% | 0% |
| ***24*** | 14% | 19% | 21% | 0% | 0% | 0% | 0% |
| ***25*** | 15% | 19% | 21% | 0% | 0% | 0% | 0% |
| ***26*** | 24% | 29% | 21% | 0% | 0% | 0% | 0% |

Table S2 Detailed vaccination coverage assumptions in males by age and calendar years considered in baseline analysis

|  | ***Calendar year*** | | | |
| --- | --- | --- | --- | --- |
| ***Age (years)*** | ***2013*** | ***2014*** | ***2015*** | ***2016 onwards*** |
| ***12*** | 75% | 76% | 76% | 76% |
| ***13*** | 0% | 0% | 0% | 0% |
| ***14*** | 66% | 72% | 0% | 0% |
| ***15*** | 32% | 0% | 0% | 0% |
| ***16*** | 1% | 0% | 0% | 0% |

# Test accuracy assumptions

Test accuracy for conventional cytology were calibrated to observed call rates and the correlation between high-grade cytology and histology outcome under the pre-renewed NCSP.[[9](#_ENREF_9)] Test accuracy for manually-read LBC was derived by calibrating to the pooled relative test-sensitivity and specific between manually-read LBC and conventional cytology reported by an international meta-analysis.[[10](#_ENREF_10)] The test accuracy of HPV testing were derived d based on data from another international meta-analyses on the test performance of HPV testing and the relative performance of HPV test compared to cytology testing.[[11](#_ENREF_11)] The test accuracy of colposcopy was based on a large colposcopy dataset (over 21,000 colposcopies) supplied by the Royal Women’s Hospital in Victoria.[[12](#_ENREF_12),[13](#_ENREF_13)] These assumptions are summarised in Table 1 in the main text and have been described in detailed previously. [[1](#_ENREF_1)]

# Population size assumptions

Model predicted age-standardised rates have been standardised to the 2001 Australian Standard Population as released by the Australian Bureau of Statistics. [14]

Table S3 Estimated 2001 Australian female population

| Age group (years) | 2001 Australian Standard Population  (2001) [14] |
| --- | --- |
| 0-4 | 1,282,357 |
| 5-9 | 1,351,664 |
| 10-14 | 1,353,177 |
| 15-19 | 1,352,745 |
| 20-24 | 1,302,412 |
| 25-29 | 1,407,081 |
| 30-34 | 1,466,615 |
| 35-39 | 1,492,204 |
| 40-44 | 1,479,257 |
| 45-49 | 1,358,594 |
| 50-54 | 1,300,777 |
| 55-59 | 1,008,799 |
| 60-64 | 822,024 |
| 65-69 | 682,513 |
| 70-74 | 638,380 |
| 75-79 | 519,356 |
| 80-84 | 330,050 |

Case numbers for were calculated using the Australian Bureau of Statistics (ABS) estimates of the resident population for 2005-2012, and the ABS ‘Series B’ population projections from 2013-2035. [14, 15]

Figure S1 Population size assumptions for the Australian female population over time.


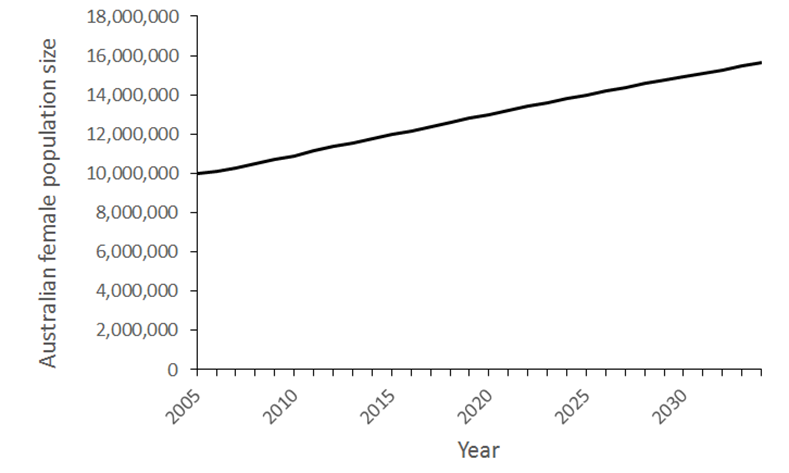


# Steady-state analysis

## Additional analysis description

The following additional analysis was carried out to clarify the impact of the renewed NCSP on steady-state (long-term) predictions for histologically-confirmed CIN2/3, cervical cancer incidence and cervical cancer mortality. Health outcomes were compared for two, single, Australian cohorts. The first cohort is assumed to be managed under the pre-renewed NCSP for the entirety of their lifetime, and the second cohort is assumed to be managed under the renewed NCSP for the entirety of their lifetime. Neither cohort is impacted by HPV vaccination. Parameter and screening program assumptions are as detailed in the main text.

## Additional analysis findings

In the absence of HPV vaccination, cohorts managed entirely under the renewed NCSP are expected to have 7% more histologically-confirmed CIN2/3 attributable to HPV types 16/18 (ASR: 1.05 per 1,000 women) than women managed entirely under the pre-renewed NCSP (ASR: 0.98 per 1,000 women), see Figure S2 (a). However, cohorts managed under the renewed NCSP are expected to have 13% less histologically-confirmed CIN2/3 attributable to HPV type not 16/18 (ASR: 0.51 per 1,000 women) than women managed entirely under the pre-renewed NCSP (ASR: 0.58 per 1,000 women), see Figure S2 (b).

**Figure S2 Age-specific rate of histologically-confirmed CIN2/3 attributable to HPV types 16/18 (a) and HPV types not 16/18 (b) for two unvaccinated cohorts.**


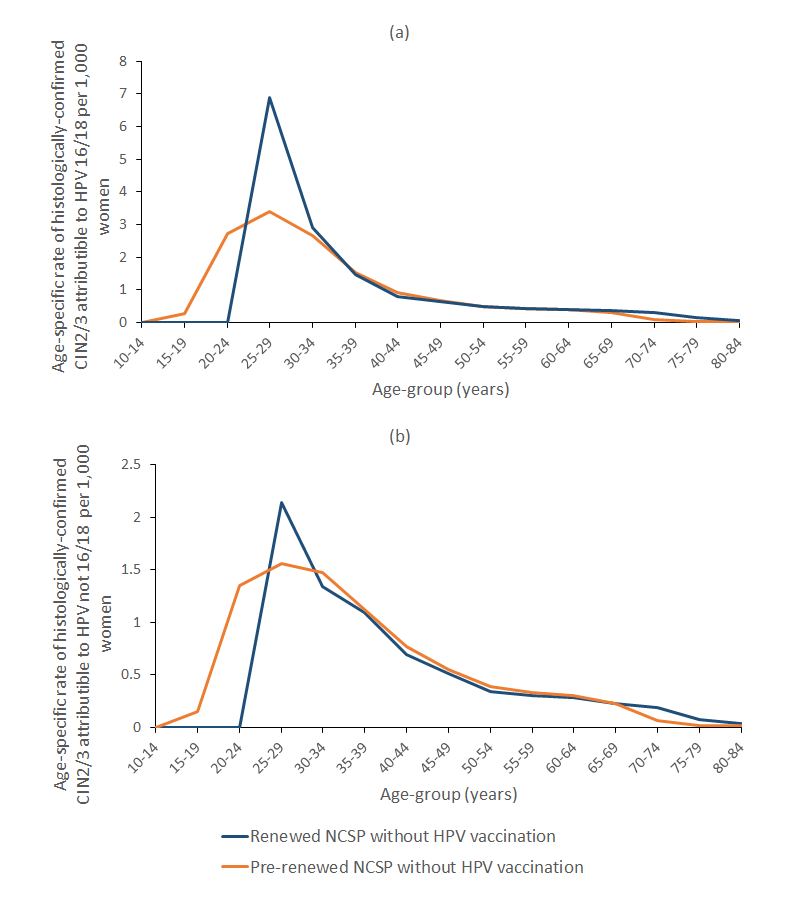


Steady-state age-standardised rates of cervical cancer incidence attributable to HPV types 16/18 for cohorts managed under the renewed NCSP (ASR: 3.45 per 100,000 women) are predicted to be 36% lower than for cohorts managed under the pre-renewed NCSP (ASR: 5.36 per 100,000 women). Similarly, age-standardised rates of cervical cancer incidence attributable to HPV types 16/18 for cohorts managed under the renewed NCSP (ASR: 1.28 per 100,000 women) are predicted to be 18% lower than for cohorts managed under the pre-renewed NCSP (ASR: 1.56 per 100,000 women). Age-specific cervical cancer incidence rates are displayed in Figure S3.

Figure S3 Age-specific rate of cervical cancer incidence attributable to HPV types 16/18 (a) and HPV types not 16/18 (b) for two unvaccinated cohorts.


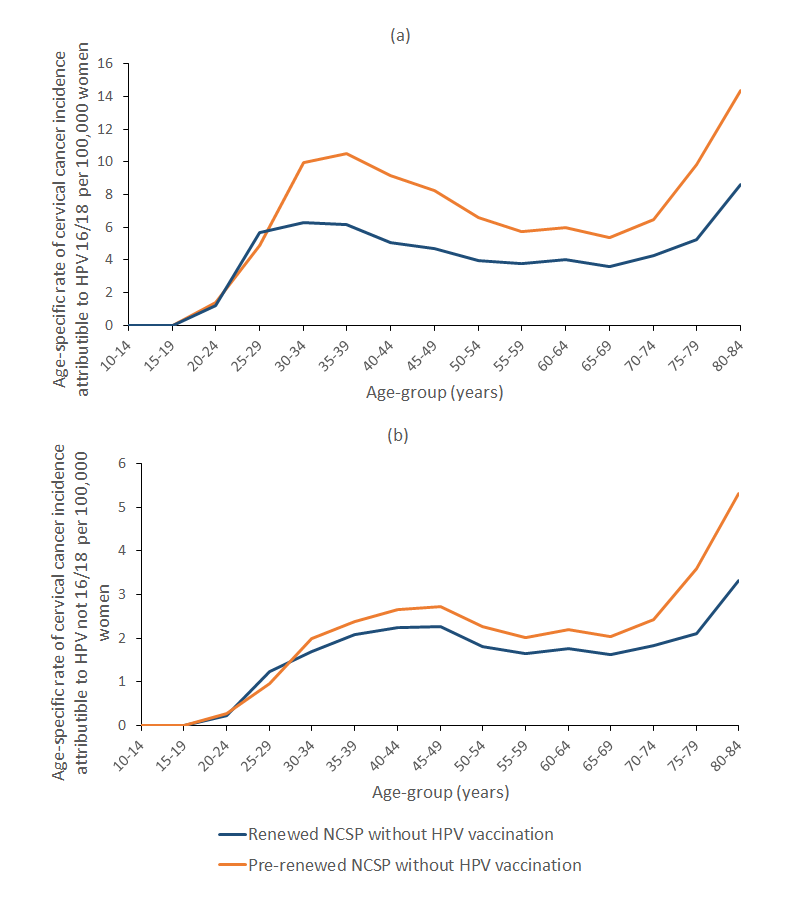


Finally, the age-standardised rate of cervical cancer mortality for unvaccinated cohorts managed under the renewed NCSP (ASR: 1.15 per 100,000 women) is predicted to be 38% lower than for cohorts managed under the pre-renewed NCSP (ASR: 1.87 per 100,000 women). Age-specific cervical cancer mortality rates are displayed in Figure S4.

Figure S4 Age-specific rate of cervical cancer mortality for two unvaccinated cohorts.


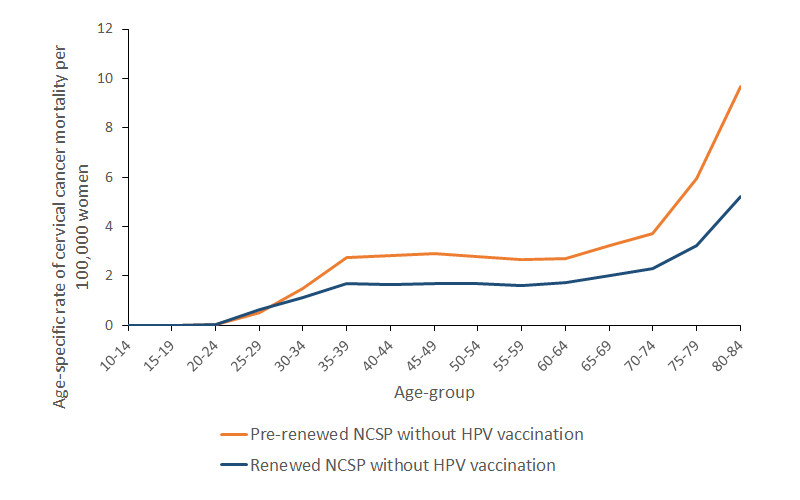


# References

1. Lew JB, Simms K, Smith MA, Kang YK, Xu XM, Caruana M, et al. (2014) National Cervical Screening Program Renewal: Effectiveness modelling and economic evaluation in the Australian setting (Assessment Report). MSAC application number 1276. Canberra: Department of Health

2. National Health and Medical Research Council (2005) Screening to Prevent Cervical Cancer: Guidelines for the Management of Asymptomatic Women with Screen Detected Abnormalities. Canberra: Commonwealth of Australia.

3. Cancer Council Australia Cervical Cancer Prevention Guidelines Working Party (2016) Draft clinical management guidelines for the prevention of cervical cancer. Available at: <http://wiki.cancer.org.au/australia/Guidelines:Cervical_cancer/Prevention>. Accessed 18 February 2016.

4. Brotherton J, Deeks S, Campbell-Lloyd S, Misrachi A, Passaris I, Peterson K, et al. (2008) Interim Estimates of Human Papillomavirus Vaccination Coverage in the School-Based Program in Australia. Commun Dis Intell Q Rep 32: 457-461.

5. Brotherton J, Gertig D, Chappell G, Rowlands L, Saville M (2011) Catching up with the catch-up: HPV vaccination coverage data for Australian women aged 18-26 years from the National HPV Vaccination Program Register. Commun Dis Intell Q Rep 35: 197-201.

6. Effler P (2015) School-based HPV Immunisation Programs: The WA Experience. Preventing Cervical Cancer conference (PCC 2015), 20-22 February 2015. Melbourne

7. Brotherton JML, Liu B, Donovan B, Kaldor JM, Saville M (2014) Human papillomavirus (HPV) vaccination coverage in young Australian women is higher than previously estimated: independent estimates from a nationally representative mobile phone survey. Vaccine 32: 592-597.

8. National HPV Vaccination Program Register (2015) HPV Vaccination Coverage Data. Available at: <http://www.hpvregister.org.au/research/coverage-data>. Accessed 7 Aug 2015 2015.

9. Australian Institute of Health and Welfare (2013) Cervical screening in Australia 2010-2011. Cancer series 76. Cat no. CAN 72. Canberra: AIHW.

10. Arbyn M, Bergeron C, Klinkhamer P, Martin-Hirsch P, Siebers AG, Bulten J (2008) Liquid compared with conventional cervical cytology: a systematic review and meta-analysis. Obstetrics & Gynecology 111: 167-177.

11. Arbyn M, Ronco G, Anttila A, Meijer CJ, Poljak M, Ogilvie G, et al. (2012) Evidence regarding human papillomavirus testing in secondary prevention of cervical cancer. Vaccine 30 Suppl 5: F88-99.

12. Medical Services Advisory Committee (2009) Human Papillomavirus Triage Test For Women With Possible or Definite Low-Grade Squamous Intraepithelial Lesions. MSAC reference 39, Assessment report. Canberra: Australian Government Department of Health.

13. Medical Services Advisory Committee (2009) Automation Assisted and Liquid Based Cytology for Cervical Cancer Screening. MSAC reference 1122, Assessment report. Canberra: Australian Government Department of Health.

14. Australian Bureau of Statistics (2013) 3101.0 Australian Demographic Statistics.

15. Australian Bureau of Statistics (2013) 3222.0 Population Projections, Australia.
